# Immunity of replicating Mu to self-integration: a novel mechanism employing MuB protein

**DOI:** 10.1186/1759-8753-1-8

**Published:** 2010-02-01

**Authors:** Jun Ge, Zheng Lou, Rasika M Harshey

**Affiliations:** 1 Section of Molecular Genetics and Microbiology and Institute of Cellular and Molecular Biology, University of Texas at Austin, Austin, TX 78712, USA

## Abstract

We describe a new immunity mechanism that protects actively replicating/transposing Mu from self-integration. We show that this mechanism is distinct from the established *cis*-immunity mechanism, which operates by removal of MuB protein from DNA adjacent to Mu ends. MuB normally promotes integration into DNA to which it is bound, hence its removal prevents use of this DNA as target. Contrary to what might be expected from a *cis*-immunity mechanism, strong binding of MuB was observed throughout the Mu genome. We also show that the *cis*-immunity mechanism is apparently functional outside Mu ends, but that the level of protection offered by this mechanism is insufficient to explain the protection seen inside Mu. Thus, both strong binding of MuB inside and poor immunity outside Mu testify to a mechanism of immunity distinct from *cis*-immunity, which we call 'Mu genome immunity'. MuB has the potential to coat the Mu genome and prevent auto-integration as previously observed *in vitro *on synthetic A/T-only DNA, where strong MuB binding occluded the entire bound region from Mu insertions. The existence of two rival immunity mechanisms within and outside the Mu genome, both employing MuB, suggests that the replicating Mu genome must be segregated into an independent chromosomal domain. We propose a model for how formation of a 'Mu domain' may be aided by specific Mu sequences and nucleoid-associated proteins, promoting polymerization of MuB on the genome to form a barrier against self-integration.

## Background

Transposition is a double-edged sword, allowing elements to populate new sites within their host genomes while potentially exposing their own DNA to self-disruption. Several bacterial transposons including members of the Tn*3 *family, Tn*7 *and bacteriophage Mu display transposition immunity [1]. These elements avoid insertion into DNA molecules that already contain a copy of the transposon (a phenomenon called *cis*-immunity) and it is thought that this form of self-recognition must also provide protection against self-integration. *Cis *immunity does not provide protection to the whole bacterial genome on which the transposon is resident, but can extend over large distances from the chromosomal site where the transposon is located, or over an entire plasmid harboring the transposon.

*In vitro *studies with phage Mu provided the first molecular insights into the *cis*-immunity phenomenon [2,3]. Mu transposition requires two Mu proteins: (1) the MuA transposase, which binds specifically to the ends of Mu and catalyzes the DNA breakage and joining reactions of transposition, and (2) MuB, an ATP-dependent DNA-binding protein that directs the transpososome complex to integrate into DNA to which MuB is bound [4,5]. MuA-MuB interaction also stimulates the ATPase activity of MuB and promotes its dissociation from DNA. This latter activity has been demonstrated to be the basis of the observed transposition immunity of mini-Mu plasmids *in vitro*; that is, MuB bound to plasmid DNA dissociates upon interaction in *cis *with MuA bound to the Mu ends, resulting in MuB-free DNA, which is a poor target for new insertions [2,6]. MuB also dissociates upon interaction with MuA in *trans*, but the oligomeric state of MuA, for example, monomer when bound to ends versus multimer when assembled into an active transpososome, may distinguish interactions at the ends that underlie *cis *immunity from those that promote target capture and transposition in *trans *[6]. The mechanism of Tn*7 *target immunity is related to that of Mu. Like Mu, Tn*7 *also has an ATP-dependent target-recognizing protein, TnsC, which can control the activity of the transposase TnsAB via ATP hydrolysis [7,8].

Phage Mu uses transposition to amplify its 37 kb genome at least 100-fold during the lytic growth cycle. To produce viable progeny, Mu must avoid transposing into itself, a daunting task given that nearly half the host genome is composed of Mu sequences by the end of the lytic cycle and that Mu lacks target specificity. Target immunity *in vivo *has been demonstrated with mini-Mu plasmid substrates [9,10], and is assumed to operate within the Mu genome as well. Support for a *cis*-immunity mechanism, which would remove MuB protein from the vicinity of the Mu genome *in vivo*, came from studies using a 10 kb derivative of Mu (Mu*d*), which was monitored for transposition into Tn*10 *elements placed at various distances from the Mu*d *element on the *Salmonella typhimurium *chromosome [11]. A gradient of insertion immunity was observed in both directions from the Mu*d *insertion point, insertion being unobstructed when the separation between the Tn*10 *target and Mu*d *was 25 kb, but undetectable when the separation was 5 kb. Immunity decayed more sharply in a gyrase mutant than in a wild-type strain, leading to a proposal that supercoil diffusion promotes transposition immunity [11]. These experiments monitored insertion immunity outside the Mu ends and not within the Mu genome.

A different form of immunity against self-integration is observed with retroviruses such as Moloney leukemia virus (MLV) and HIV, which protect their DNA against intramolecular insertion by using a protein called barrier to auto-integration factor (BAF) [12,13]. BAF, a dimeric protein and a cellular component of viral pre-integration complexes, bridges viral DNA non-specifically and condenses it [14,15]. BAF appears to play a dual role, compacting DNA reversibly to prevent auto-integration on the one hand, while promoting intermolecular target capture on the other [16]. MuB has the potential to provide a BAF-like immunity mechanism, as it can polymerize non-specifically on DNA [17]. MuB has a preference for AT-rich DNA [18] and was observed to bind strongly to synthetic A/T-only DNA, where it probably formed a continuous filament [19]. The MuB-bound region was refractory to integration. Although MuB is not expected to bind stably in the interior of the Mu genome because the A/T content of this region is low [20], A/T content is not a reliable predictor of Mu behavior *in vivo*. Higher binding of MuB was observed to hot versus cold gene targets, even though the hot genes had an average A/T content and cold genes had higher A/T values [19]. MuB binding is expected to be modulated by host proteins *in vivo*.

In this work we set out to determine if the Mu genome is indeed immune to self-integration during replicative transposition, and if so, to test whether this is due to the *cis*-immunity mechanism. Operation of such a mechanism predicts that MuB would be cleared from the interior of the Mu genome (that is, bound poorly there), although MuB might also bind poorly because of high transcription through Mu or because of the low A/T content of its genome, both of which are expected to disfavor binding [20-22]. We show that the Mu genome is indeed fairly refractory to self-insertion *in vivo*, but that contrary to expectation, MuB binds strongly within Mu. Our data show that this immunity is restricted to the Mu genome and that the *cis*-immunity mechanism is apparently operative immediately outside the Mu ends. We call this new mechanism of immunity 'Mu genome immunity'.

## Results

### The Mu genome is immune to integration *in vivo*

**Figure 1:**
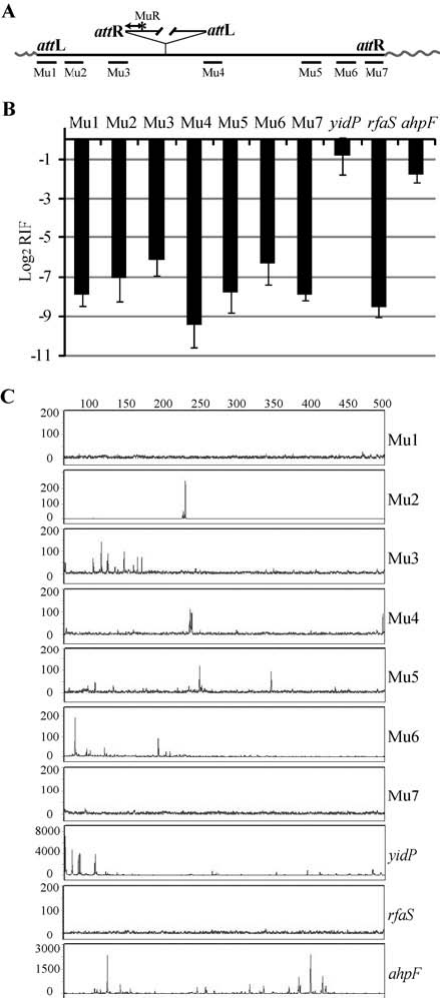
**Quantification and profile of Mu insertions within Mu *in vivo***. **(a) **Experimental strategy. A common MuR primer (labeled* with 6FAM for experiments in **(c)**), anneals within the right end or *att*R of Mu. Primers annealing to seven different locations within Mu (Mu1 to Mu7) were each paired with MuR in PCR reactions to measure **(b) **the frequency and **(c) **the site of Mu insertions packaged in phage particles. Wavy lines indicate host DNA linked to Mu ends in packaged phage heads. **(b) **Real-time PCR reactions were performed using Mu DNA purified from phage prepared after induction of strain SJG3. Ct values are inversely proportional to the amount of nucleic acid of interest in the sample. Log_2 _of the relative insertion frequency (RIF) values were derived from Ct differences between samples and input Mu DNA control; primer pairs annealing within region 4 served as controls for input DNA. Primers hybridizing to *E. coli *genes known to be hot (*yidP*), cold (*rfaS*) and average (*ahpF*) Mu insertion targets were also paired with MuR as controls. Primer efficiencies were calculated as described in Methods. The data are an average of three technical repeats. (**c**) Profile of Mu insertions within the targets monitored in (**b**). PCR reactions were as in (**b**), except that MuR was labeled with the fluorescent primer 6FAM. The reactions were subjected to FLA. Numbers on the X axis refer to nucleotides. The intensity of the fluorescent signal reflects the frequency of insertion at a particular site and is represented by arbitrary numbers on the Y axis.

To test if Mu is immune to self-integration, we monitored Mu integration within itself by analyzing DNA in Mu virions as follows. Mu packages its DNA by a head-full mechanism starting at the left or *att*L end until approximately 40 kb of DNA has been packaged [23]. A segment of the inserted Mu copy is therefore expected to be packaged in the genome of the recipient (Figure 1a). Insertions across representative regions within the Mu genome were analyzed by PCR, employing a common primer hybridizing to the right or *att*R end and a second primer within the Mu region being tested. This method will detect insertions whose R end is oriented towards the L end of the recipient in which packaging initiates; the R end of an oppositely oriented insertion would lie beyond the head-full size of ~40 kb. Although some bias has been reported at the local level [24,25], Mu insertions in the *Eschericia coli *genome generally show no orientation bias at the level of W/C strands; we believe that this is true of insertions within Mu as well and that the data are representative of all insertions. This method will report on both inter- and intra-molecular insertions. As controls, we included three known 'hot', 'cold' and 'average' targets of Mu - *yidP*, *rfaS *and *ahpF*, respectively - [19,22], whose DNA would be linked to the R end in a packaged genome.

Quantitative PCR (qPCR) was performed to analyze the frequency of insertions within seven regions spanning Mu (Figure 1b). These are plotted as relative insertion frequency (RIF) values, which represent cycle threshold (Ct) differences between the sample and input Mu DNA control. Lower RIF values mean lower insertion frequency in the target. RIF values of the hot, cold and average Mu targets were consistent with expectation. Insertions within all seven regions of Mu had RIF values similar to the cold target *rfaS*.

A parallel set of PCR reactions were performed with the MuR primer fluorescently labeled with 6FAM and subjected to Fragment Length Analysis (FLA) to monitor insertion profiles within a 500 bp region in each target (Figure 1c). The heights of the peaks represent the relative frequency of insertion. Insertions into the end regions 1 and 7 of Mu resembled those in *rfaS*. These regions harbor MuA binding sites, and the transpososome assembled here is expected to prevent access to this DNA. The internal regions 3 to 6 showed a handful of insertions with peak heights of approximately 100. These values were much lower than those of the majority of insertions in *ahpF *and *yidP*, which had peak heights of > 500, with many insertions showing peak heights up to 4000.

Data in Figure 1b are derived from the qPCR method, which measures DNA amounts based on the fluorescence signal from SYBR-bound DNA. We assume that Mu insertions are distributed evenly in the regions tested. Data in Figure 1c are derived from hybridization of a labeled primer and directly reflect the molar amounts of insertions present. The conformity of these two different kinds of measurements increases our confidence in the data. We conclude that the Mu genome is fairly, but not entirely, immune to integration.

### The Mu genome is not immune to integration *in vitro*

**Figure 2:**
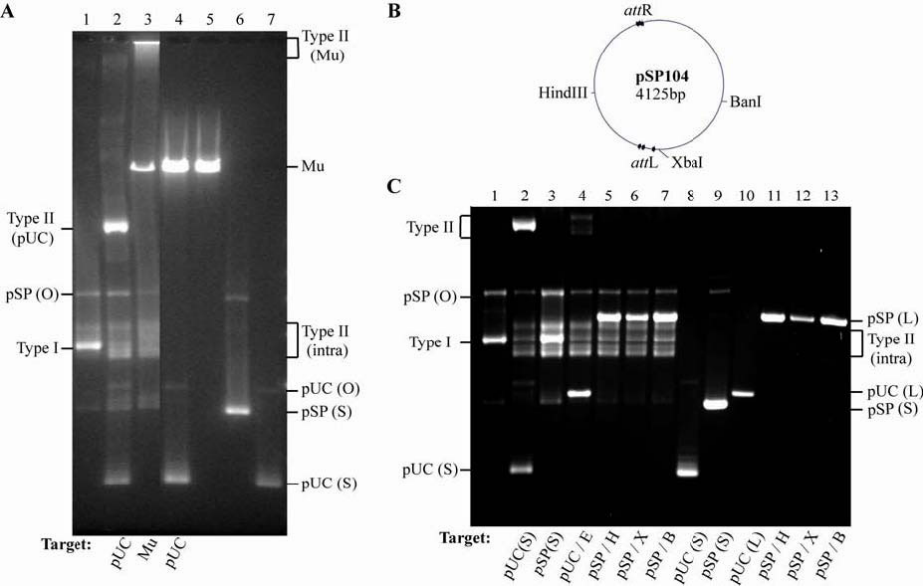
**Immunity of the linear Mu genome *in vitro***. (**a**) Transposition reactions were set up by incubating MuA, MuB and HU proteins with the mini-Mu plasmid pSP104 as donor and either linear Mu genome or pUC19 as target, as described in Methods. Lane 1, cleaved Type I complex assembled on pSP104; lanes 2 to 4, Type II or strand transfer reactions with: pSP104 as donor and either pUC19 (lane 2) or Mu (lane 3) as target or Mu as donor and pUC19 as target (lane 4); lanes 5 to 7, control substrates without added proteins. (**b**) Map of pSP104 showing restriction enzyme sites used for linearization and their position with respect to the *att*L and *att*R Mu ends. (**c**) Transposition reactions with linear mini-Mu as target. Reaction conditions were as in (**a**), except that Type I complexes were first assembled on pSP104 and added to indicated targets in a second step. Lane1, Type I reaction; Lane 2 to 7, Type II reactions. Lanes 8 to 13, DNA controls without added proteins. Restriction enzyme shown in the B panel are abbreviated to H, X and B. L = linear. O = open circular; S = supercoiled; Type I = cleaved complex; Type II = strand transfer complex; Type II (intra) = intramolecular strand transfer complexes.

Whereas it is known that mini-Mu plasmids are immune to integration and are not used as targets *in vitro*, the immunity of the whole Mu genome has not been tested *in vitro*. We compared the efficiency of use of a mini-Mu plasmid versus the phage Mu genome as target in transposition reactions employing MuA and MuB proteins and the mini-Mu donor plasmid pSP104 (Figure 2a). When incubated with MuA, pSP104 forms a complex called Type I, wherein Mu ends have undergone single-strand cleavages (lane 1). Inclusion of a target plasmid (pUC19) and MuB results in efficient integration of pSP104 into pUC19 to give the intermolecular Type II strand transfer complex (lane 2). (The smear of bands around the Type I complex represent intramolecular transposition [26,27]). In reactions using pSP104 as donor and the Mu genome as target, the Type I complex was efficiently consumed along with substantial consumption of the linear Mu genome target, resulting in the appearance of intermolecular Type II strand transfer products of large molecular weight (lane 3). The linear Mu genome did not itself serve as a donor as shown by absence of any reaction when pUC19 was provided as target (lane 4). A linear donor substrate is not expected to undergo cleavage at Mu ends, because supercoiling is essential for assembling the transpososome on the paired Mu ends under these assay conditions [5].

An important difference between the Mu genome substrate *in vivo *versus that employed *in vitro *is that Mu exists as part of the supercoiled *E. coli *genome *in vivo*, but is linear when isolated from virions. If lack of immunity of the Mu genome *in vitro *is due to its linear configuration, a linear mini Mu plasmid should also not display immunity; that is should serve as target. Three different restriction enzymes sites were used to linearize pSP104 (Figure 2b) to produce different lengths of potential target DNA flanking the Mu ends. Reactions with linearized plasmid pSP104 and linearized control pUC19 are shown in Figure 2c. Type I complex was first assembled on pSP104 (lane 1), and the cleaved complex was subsequently added to various supercoiled and linear targets. Supercoiled pUC19 was used efficiently as target, giving the Type II complex (lane 2), whereas supercoiled pSP104 was simply converted to more Type I and did not serve as target (lane 3). Linear pUC19 was used as a target, but at a lower efficiency (lane 4), whereas linear pSP104 was not used as target, irrespective of the enzyme used for linearization (lanes 5 to 7).

In summary, a linear mini-Mu is immune to integration *in vitro*, but a linear Mu genome is not.

### Insertions within linear Mu *in vitro *are directed to regions away from the ends

**Figure 3:**
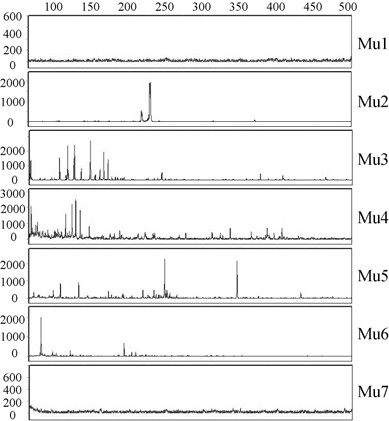
**Profile of Mu insertions within the Mu genome *in vitro***. Reactions were as described in Figure 1c, except that the template for PCR was the *in vitro *Type II reaction shown in Figure 2, lane 3.

Insertions of mini-Mu into linear Mu obtained in the transposition reaction shown in Figure 2 (lane 3), were analyzed with the same primer pairs used to analyze insertions into the Mu genome *in vivo *(Figure 1c). The results are shown in Figure 3. Insertions were absent only at the MuA binding sites near the Mu ends (region 1 and 7). The end-proximal regions 2 and 6 had fewer insertions compared with the central regions 3 to 5. This pattern of insertions away from the Mu ends is consistent with the *cis*-immunity mechanism clearing MuB near the vicinity of MuA-bound ends.

### MuB binds throughout the Mu genome *in vivo*

From the results shown in Figures 1 to 3, it is clear that the Mu genome is immune *in vivo*, but the *cis*-immunity mechanism is not sufficient to explain this immunity because it does not protect the internal regions of Mu *in vitro*. An indicator of an operative *cis*-immunity mechanism *in vivo *would be absence of MuB binding within the Mu genome. In chromatin immune precipitation (ChIP) experiments using MuB antibodies, the PCR signal was not sufficiently strong, so we engineered a c-myc epitope tag at the N-terminus of MuB on the prophage genome (strain SJG3). MuB is essential for Mu replication. c-myc-MuB supports normal Mu replication as judged by normal lysis profiles and phage titers obtained after induction of prophages carrying normal or c-myc-MuB [see Additional file 1, comparing MP1999 to SJG3]. In addition, Mu insertion profiles within the hot *E. coli *target *yidP *were similar in strains with either wild-type MuB [19] or c-myc-MuB (compare *yidP *data from Figure 1 in this study to Figure 3 in reference [19]). Thus, c-myc-MuB is functionally indistinguishable from wild-type MuB. Other tags (His and GFP) at the N-terminus of MuB have been shown to retain all MuB functions tested *in vitro*, including *cis *immunity [6,18].

**Figure 4:**
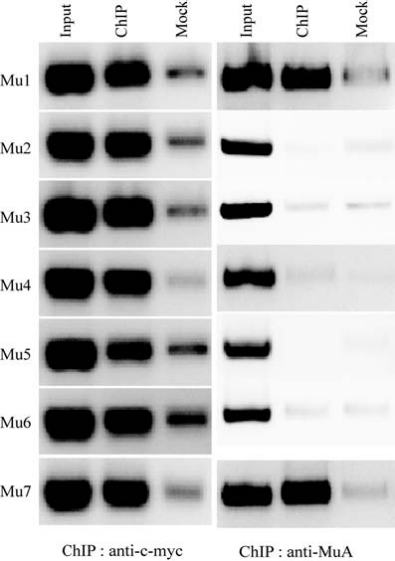
**ChIP reactions probing binding of c-myc-MuB and MuA on the Mu genome during Mu replication**. ChIP samples were prepared with either anti-c-myc antibody or anti-MuA antibody using strain SJG3 as described in Methods. Binding on the seven different segments of Mu genome shown in Figure 1a was tested by regular PCR amplification. Input = fragmented whole genome DNA; ChIP = DNA in ChIP samples; Mock = DNA recovered without addition of specific antibody during the ChIP procedure.

Mu replication was induced in SJG3 by inactivating the temperature-sensitive Mu repressor at 42°C. Western blots showed MuB levels increasing continuously after induction of Mu replication [see Additional file 1]. Cells were harvested at 40 minutes after induction to ensure both an actively replicating Mu population and adequate MuB levels. The average Mu copy number was calculated to be 18 at this time point (see Methods). ChIP samples prepared using c-myc antibody were probed for DNA spanning the seven regions within Mu (Figure 1) by regular PCR reactions using primer pairs within these regions. Mock controls without antibody were used to assess the contribution of non-specific binding. MuB was observed to bind strongly to all seven regions examined (Figure 4). As a control, we also conducted ChIP experiments with anti-MuA antibody in cultures harvested in parallel. MuA binds to specific sites at the L and R ends, but is also known to have non-specific DNA-binding activity [5,28]. In reaction conditions equivalent those in the MuB ChIP samples, MuA binding was detected only at the ends and not to internal regions. Because we know that site-specific binding of MuA to Mu ends is strong, especially in the context of an assembled transpososome, we interpret the equivalent MuB signal across all regions of the Mu genome to represent strong binding. This result is contrary to that expected of the *cis*-immunity mechanism [2].

### Strong MuB binding is confined to the interior of the Mu genome

**Figure 5:**
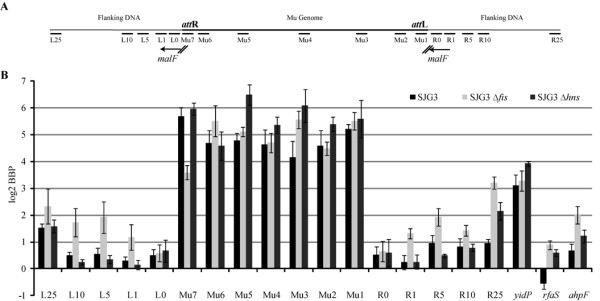
**qPCR of DNA isolated by ChIP using anti-c-myc-MuB antibody after induction of Mu replication**. (**a**) Location of DNA segments within and outside the Mu prophage. The Mu insertion is in *malF*, whose direction of transcription on the *E. coli *genome is indicated by arrows. The L0 region spans 45 to 328 bp from the beginning of *att*R, whereas the R0 region spans 89 to 343 bp from the beginning of *att*L. L1 to L25 and R1 to R25 indicate approximate distance in kb from the Mu ends. Mu1 to Mu7 amplify the following regions in bp, the numbering starting at 1 on the *att*L. Mu1: 1 to 350; Mu2: 2,650 to 3,021; Mu3: 9,915 to 10,245; Mu4: 17,641 to 17,983; Mu5: 26,419 to 26,790; Mu6: 34,223 to 34,660; Mu7: 36,421 to 36,717. See Additional file 3 for primer sequences. (**b**) MuB binding to regions indicated in A in three Mu lysogen strains: SJG3, SJG3 *Δfis *and SJG3 *Δhns*. Real-time PCR reactions of ChIP samples and Mu copy number estimates were performed as described in Methods. MuB binding to hot (*yidP*), cold (*rfaS*) and average (*ahpF*) Mu insertion target genes in *E. coli *was monitored in parallel (see Figure 1). The log_2 _BBP values are the Ct difference between ChIP and mock samples for each segment. The data are an average of nine experiments: three independent biological repeats, each with three independent technical repeats. BBP = MuB binding preference.

Real-time PCR was used to compare MuB binding across Mu and to chromosomal DNA flanking the original Mu insertion in the ChIP samples (Figure 5a). Control hot (*yidP*) and cold (*rfaS*) Mu targets tested for MuB binding in an earlier study were also used [19], along with average target *ahpF *(Figure 1). In this earlier study conducted with anti-MuB antibody, the hot target *yidP *bound MuB more strongly than did the cold target *rfaS*. Similar results were seen in this study with anti-c-myc antibody (black bars, SJG3). Within the Mu genome, MuB binding was uniformly high throughout, even though the genome is as cold a target as *rfaS *(Figure 1b). These values, when corrected for Mu copy number, could be an underestimate if MuB binds only to a subset of Mu genomes that have finished replication.

To determine how the binding pattern of MuB within the Mu genome compares with that in its chromosomal vicinity, the ChIP samples were amplified using primer pairs annealing to known genes flanking the original Mu insertion. DNA on both sides of the insertion at ~300 bp, 1 kb, 5 kb, 10 kb and 25 kb from the ends was monitored (L/R 0 to 25, respectively; black bars, SJG3). Binding at L0 to L10 was lower than at L25, and binding at R0 to R1 was lower than at R5 to R25.

In summary, strikingly different patterns of MuB binding were seen inside and immediately outside the Mu genome. Outside the Mu ends, increased binding away from the ends could be discerned, consistent with a *cis*-immunity mechanism. Inside Mu, the uniformly high MuB binding suggests a different mechanism, which operates in the presence of MuB binding.

### Chromosome-organizing proteins Fis and H-NS affect MuB binding

The clear distinction in MuB binding patterns between Mu and non-Mu sequences suggests that there exists a physical boundary between these regions. We therefore considered whether nucleoid-organizing host proteins could be involved in creating a separate 'Mu domain', which promotes MuB binding. The rationale for this came from the longstanding observation that mutations in H-NS or Fis, proteins that participate in compacting the *E. coli *nucleoid [29], de-repress a Mu prophage, indicating their likely presence on the Mu genome [30-32]. We therefore generated Δ*fis *and Δ*hns *mutations in the SJG3 strain, and monitored MuB binding to the Mu and flanking regions as before. A Δ*fis *Δ*hns *double mutant was not viable in this background.

Outside the Mu ends, MuB binding increased significantly at a majority of sites in the *fis *mutant (Figure 5). The *hns *mutation had a similar effect only at R25, the overall tendency in this mutant being to decrease MuB binding at most sites. Fis, H-NS and MuB all prefer A/T-rich DNA and probably compete for similar binding sites. Because Fis is the most abundant nucleoid-associated protein in the cell [33], the increase in MuB binding in the *fis *mutant could be due to availability of sites previously occupied by Fis. Despite the altered MuB binding patterns in these two mutants, the gradient of increased MuB binding away from the ends was maintained. Such a gradient was strikingly absent inside the Mu ends, although binding did increase at some locations, primarily in the *hns *mutant. The cold site *rfaS *showed increased MuB binding in both mutants, whereas the average site *ahpF *showed increased binding only in the *fis *mutant and the hot site *yidP *increased only in the *hns *mutant.

In summary, the absence of Fis or H-NS changes MuB binding profiles on DNA. Despite the changed profiles, the gradient of increasing MuB binding away from the ends is maintained outside Mu, whereas uniform binding is maintained inside Mu.

### The Mu genome is far more immune than its flanking DNA

**Figure 6:**
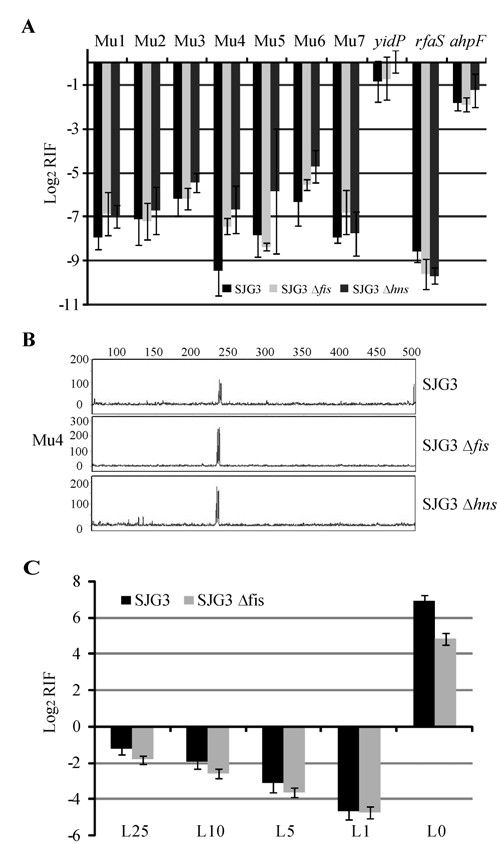
**Mu insertions within Mu and flanking DNA in *fis *and *hns *mutants**.(**a**) Data for SJG3 are from Figure 1b. All other descriptions as in Figure 1b. (**b**) FLA analysis of reactions for the Mu4 region. Other descriptions as in Figure 1c. (**b**) As in (a), except insertions were monitored in L0 to L25 regions in the indicated strains.

To test whether the change in MuB binding would change Mu insertion profiles, qPCR reactions were conducted for all the Mu segments and control targets in the *fis *and *hns *mutants, as well as for segments L0 to L25, where increased MuB binding was observed in the *fis *mutant compared with the wild type. Within Mu, a small increase in Mu insertions was observed only in the central region 4 in both *fis *and *hns *mutants (Figure 6a), although MuB binding did not alter substantially in this region (Figure 5b). FLA analysis showed that insertion at a specific site around 240 bp in this region showed a moderate increase in both mutants (Figure 6b). Thus, protection of the Mu genome is largely maintained in these mutants. Interestingly, despite increased MuB binding, the cold site *rfaS *became slightly colder in both mutants (Figure 6a and Figure 5b), but the hot site *yidP *became slightly hotter in the *hns *mutant. Taken together, these data show that MuB binding makes some regions cold and other regions hot. Therefore, some other cellular feature must modulate MuB activity to generate the opposing Mu insertion outcomes.

Outside Mu, in segments L1 to L25, a gradient of Mu insertions was observed in the wild-type SJG3 strain, being lowest at L1 and highest at L25, consistent with the *cis*-immunity mechanism (Figure 6c). Despite the increased MuB binding to this region in the absence of Fis (Figure 5), the Mu insertion frequency did not change significantly in the *fis *mutant compared with the wild type. The signal at L0 is high because the original insertion in *malF *is expected to be packaged in around 4% of phage particles [34].

When insertion patterns inside and outside Mu are compared, a difference of at least an order of magnitude is observed, the inside being more refractory (compare Figure 6a to 6c). For example, in regions close to the Mu end, there are 15 times more insertions outside at L1 (1 kb) than at an equivalent distance inside at Mu1-2 (0-3 kb). A similar difference is seen if one compares L10 (10 kb) outside to Mu3 (10 kb) inside. At L25 outside, the difference in insertion frequency compared with the center of Mu (Mu4: ~18 kb) approaches 100-fold. Thus, assuming that the *cis *immunity mechanism is operative outside, it clearly does not provide the level of protection seen inside.

In summary, both MuB binding patterns and Mu insertion frequencies show that different immunity mechanisms must operate within and outside Mu.

## Discussion

This study has revealed a new immunity mechanism we call Mu genome immunity, which protects replicating Mu from self-integration. This mechanism is distinct from *cis *immunity, which appears to be functional immediately outside Mu. Sharply different patterns of MuB binding and insertion within and outside Mu on the *E. coli *chromosome indicate the existence of a mechanism for discriminating between the two regions. We propose that Mu genome immunity is enabled by segregating Mu into a separate chromosomal domain.

### Cis immunity versus Mu genome immunity

MuB has been known thus far to promote, not prevent, Mu integration on natural DNA substrates. MuB is essential both for intermolecular transposition and for *cis *immunity *in vitro *[2,3]. It seems that *cis *immunity also operates outside the Mu ends *in vivo *as inferred from the observed gradient of increasing Mu insertions in both directions from a Mu*d *insertion on the *S. typhimurium *chromosome [11], and from the results of this study on the *E. coli *chromosome where higher MuB binding was observed away from the ends (Figure 5b), along with an increasing gradient of Mu insertions (Figure 6c).

In contrast to the pattern of MuB binding outside Mu ends, strong and uniform binding was observed inside Mu along with uniform protection from insertion (Figures 1 and 4). Whereas MuB might be expected to bind strongly to the end regions 1 and 7 because it is associated with transpososomes assembled on the ends [35,36], MuB is not expected to be bound inside if the *cis*-immunity mechanism were operative here [2].

An interesting property of MuB is its proclivity to polymerize cooperatively and non-specifically on DNA, with a tendency to form larger polymers on A/T-rich DNA [17,37]. On natural DNA, which can range from 40% to 70% A/T, analysis of MuB binding and Mu insertion patterns is consistent with a fairly interspersed pattern of MuB binding, with insertions being directed to adjacent DNA sites free of MuB [19]. The pattern of Mu insertion within such DNA is unperturbed over a wide range of MuB concentrations. On the other hand, MuB binds strongly to synthetic DNA that is 100% A/T, protecting the bound DNA even at low MuB concentrations [19]. MuB binding could therefore theoretically protect the Mu genome against Mu insertion if it were to form a tightly-bound filament on it. The barrier to integration presented by a continuous MuB filament offers an alternative and antithetical (to the *cis*-immunity) mechanism to explain Mu genome immunity. However, the Mu genome is devoid of features known to promote stable MuB binding *in vitro*. Only the Mu ends are A/T-rich, and there is a gradient of high to low A/T from the ends to the center of Mu (Additional file 2). Thus, some feature other than A/T content is responsible for strong MuB binding here.

### Nucleoid-associated proteins and Mu transposition

The bacterial genome is folded into a compact structure called the nucleoid. Many architectural proteins associate with the nucleoid and contribute to the folding and compaction by bridging, bending or wrapping DNA, which is organized into many supercoiled domains [38,39]. There are estimated to be 50 to 400 supercoiled DNA loops, which are on average about 10 kb in size [39-41]. These DNA loops are discrete chromosomal territories that are topologically independent and dynamic, and are maintained by the activity of the nucleoid-associated proteins (NAPs), many of which bind to A/T-rich DNA. AT-rich sequences are also found at the ends and center of the Mu genome (Additional file 2), suggesting that Mu may form an independent chromosomal domain, aided by the binding of NAPs.

Of the dozen or so NAPs found in *E. coli *[42], most research has focused on the abundant proteins H-NS, Fis, HU and integration host factor (IHF). In contrast to eukaryotes, in which protein-mediated compaction is attributed exclusively to histones, none of these proteins is solely responsible for DNA compaction. Bacterial cells deficient in one of the NAPs usually have subtle phenotypes, which indicate that (some of) the roles of one NAP can be fulfilled by another. Double mutations of HU/Fis or HU/IHF often have more severe effects. In this study, we tested the contribution of H-NS and Fis to MuB binding and Mu genome immunity because these proteins have been shown to play a role in the stable maintenance of a Mu prophage in *E. coli *[30-32]. Fis is the most abundant protein in *E. coli *[33]. H-NS is a transcriptional repressor for many genes and has been shown to bind and silence 'foreign' DNA in *Salmonella *[43]. It can bridge two DNA helices, a property thought to aid in compacting the nucleoid, and has been demonstrated to stabilize the Tn*10 *transpososome by interacting with both DNA and the transposase [44].

In the absence of either H-NS or Fis, MuB binding was altered on the DNA regions outside the Mu ends, being more pronounced in the *fis *mutant (Figure 5). A general increase in MuB binding was also observed inside Mu in both mutants. However, both regions maintained the same level of insertion immunity as the wild-type strain (Figure 6). Representative hot and cold genes located away from Mu showed opposite behaviors in response to MuB changes. With increased MuB binding, the cold gene *rfaS *got colder whereas the hot gene *yidP *got hotter. These results suggest that MuB binding patterns are influenced by host NAPs, but that these patterns are not the sole determinants of Mu insertion frequencies. Some other cellular feature overrides the known properties of MuB and alters its activity.

### A model for Mu genome immunity

**Figure 7:**
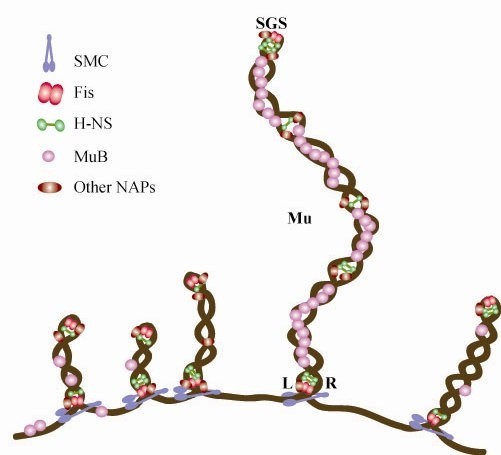
**A model for Mu genome immunity. The model proposes that segregation of Mu into a separate domain on the *E. coli *chromosome is aided by the centrally located SGS, which initiates loop formation, and is sealed by either the Mu transpososome assembled on the ends or NAPs**. Several NAPs are shown stabilizing this structure, hypothesized to promote formation of MuB filaments, which provide a barrier against self-integration. Fis and H-NS may be expected to reside at the SGS and Mu ends because these proteins prefer A/T rich regions (see Additional file 2). SMCs have been proposed to be involved in the creation of large topological loops by bridging two DNAs at the base of the stem of such loops [29,39].

Mu ends define a boundary separating two modes of MuB binding and immunity. We propose that Mu genome immunity arises from a special structure that Mu adopts, aided by both specific Mu sequences and by general cellular NAPs (Figure 7). In the center of the genome is the strong gyrase-binding site (SGS), which is essential for Mu replication *in vivo *and is believed to function by influencing efficient synapsis of the Mu ends [45-48]. The SGS is thought to act by localizing the 37 kb Mu prophage DNA into a single loop of plectonemically supercoiled DNA upon binding of DNA gyrase to the site. The SGS, predicted to reside at the turnaround of the loop, would constrain the two Mu ends to the same supercoiled domain to facilitate synapsis. We propose that an SGS-generated Mu loop, sealed off at the Mu ends by either the transpososome or NAPs, serves as a scaffold for nucleating MuB filaments in the Mu interior, providing a barrier to Mu integration. We draw parallels between Mu genome immunity and the immunity conferred by the BAF protein in HIV and MLV, which, along with other cellular proteins, compact the retroviral genomes and prevent auto-integration [49,50]. Whereas MuB has not been demonstrated to bridge two DNA helices like other DNA compacting proteins, DNA binding has been observed in both domains of this bi-domain protein [51,52]. Single molecule experiments with MuB have been carried out with DNA held in an extended conformation in a flow cell, a condition not expected in natural chromosomal DNA [53]. However, MuA- and MuB-dependent DNA looping has been observed when the flow of buffer was relaxed in this device [6]. DNA looping was also observed in BAF and Fis in similar experiments, and has been proposed to be the mechanism responsible for DNA condensation by both proteins [50,54].

Our results open a new frontier for understanding Mu genome immunity, and pose many new questions. For example, what is the timing of establishment of this immunity? How does immunity to integration still allow other processes such as transcription and replication to go on, if indeed they do? Is it possible that strong Mu transcription initially disfavors integration, but that replication through the genome marks it for genome immunity?

### What is *cis *immunity good for?

Both *in vivo *and *in vitro *data presented in the study show that the *cis*-immunity mechanism is an ineffective strategy for protecting the interior of Mu. *In vivo*, the region between 1 and 25 kb outside a Mu end had 10 to 100 fold more insertions than the region within a similar distance inside (Figure 6). *In vitro*, the linear Mu genome was not protected from mini-Mu insertions, particularly in internal regions (Figures 2 and 3). If *cis *immunity is neither effective nor operative inside the Mu genome, its role seems to be limited to discouraging Mu insertions immediately next to the ends. What is the importance of such a mechanism in the life of Mu? One possibility is that, because Mu packages flanking DNA, avoiding insertions in this DNA avoids packaging orphan L or R ends, which might interfere with assembly of the transpososome on the correct Mu ends in a new host. However, the Mu synapse is assembled in a highly ordered manner on three sites (Mu ends and an internal enhancer site) [55,56], and is topologically unique [57-59], a design that should inherently exclude incorrectly oriented 'extra' ends [60]. A second possibility is that discouraging use of nearby targets increases sampling of other regions of the chromosome, because some insertion locations are difficult to transpose out of [61]. A third possibility is that *cis *immunity is simply a byproduct of biochemical properties of MuB and MuA and has no real relevance *in vivo*.

## Conclusions

A new immunity mechanism we call Mu genome immunity protects actively replicating/transposing Mu from self-integration. This mechanism is associated with strong MuB binding within the Mu genome. The *cis*-immunity mechanism, which requires removal of MuB from DNA adjacent to Mu ends, is apparently functional immediately outside Mu, but the level of protection offered by this mechanism is insufficient to explain the protection seen inside. The sharply different patterns of MuB binding inside and outside Mu suggest that the Mu genome is segregated into an independent chromosomal domain. We propose a model for how formation of an independent 'Mu domain' might nucleate polymerization of MuB on the genome, forming a barrier against self-integration. We speculate that Mu genome immunity might be functionally similar to the immunity conferred by eukaryotic cellular BAF protein to HIV or MLV retroviral genomes. Our results also reveal that whereas MuB binding is sensitive to the presence of host nucleoid-associated proteins, MuB binding patterns are not the sole determinants of Mu insertion frequencies; some other cellular feature overrides the known properties of MuB and alters its activity.

## Methods

### Bacterial strains, DNA and proteins

*E. coli *Mu lysogen strain MP1999 [recB, recC, sbcB, *malF*::Mu*c*ts62] [46] was used to construct strain SJG3 (MP1999 Mu*B::9c-mycB*) and its two derivative mutants SJG3 *Δfis *and SJG3 *Δhns*. All deletions/substitutions were generated by the λ Red recombination system using amplification primers listed in Additional file 3[62]. In SJG3, the Mu*B *gene in the prophage is substituted with *9c-mycB*, introducing an additional 381 bp at this location. This replacement was by a two-step procedure, first introducing a *cat*-*sacB *cassette into Mu*B *in MP1999, selecting for Cam resistance, and then replacing the cassette with *9c-mycB *derived from pSJG4 selecting for sucrose resistance [63].

Plasmid pSJG4 contains *9c-mycB *cloned between *Nco*I and *Bam*HI sites of pET28a. pSP104 is a mini-Mu donor substrate with *att*L, *att*R and enhancer [57]. pUC19 was used as transposition target (laboratory stock).

MuA, MuB and HU proteins were purified as described previously [64].

### Phage purification and DNA extraction

Induction of Mu prophage and purification of phage were performed as described previously [34]. The SJG3 and SJG3Δ*fis *strains were harvested at 1.5 hours and the SJG3Δ*hns *was harvested at 3.5 hours after heat induction. Purified Mu phage suspended in Mu buffer (20 mM Tris-HCl, pH 7.5, 0.2 M NaCl, 1 mM CaCl_2_, 20 mM MgSO_4 _and 1% gelatin) was digested by pronase (0.5 mg/ml) at 37°C overnight in the presence of 20 mM EDTA (pH 8.0). After digestion, SDS was added to final concentration of 0.5% (w/v) and the mixture was incubated at 37°C for 1 hour. Mu DNA was then purified by phenol/chloroform extraction and precipitated with an equal volume of isopropanol in the presence of 0.3 M sodium acetate (pH7.0). The DNA pellet was washed twice with 75% ethanol, vacuum-dried, and dissolved in 10 mM Tris-HCl (pH 7.5).

### *In vitro *transposition

One-step strand transfer reactions contained 30 μg/ml of mini-Mu plasmid DNA, 20 μg/ml target, 10 μg/ml HU, 7 μg/ml MuA and 5 μg/ml MuB in 20 μl of 20 mM HEPES-KOH (pH 7.6), 2 mM ATP, 140 mM NaCl and 10 mM MgCl_2_. Reactions were incubated at 30°C for 30 minutes. Two-step strand transfer reactions were performed as follows. In the first step, cleaved Mu complexes were assembled at 30°C for 20 minutes as described above except without MuB and ATP; in the second step, strand transfer was initiated by adding equal amounts of the reaction mixture to tubes containing 10 μg/ml of various target DNAs, 2 mM ATP and 5 μg/ml MuB, and the reactions were incubated at 30°C for 20 minutes.

### FLA of PCR products

Mu phage DNA or *in vitro *strand transfer products were used as templates in PCR reactions with a common Mu R-end primer labeled at its 5' end with fluorescent agent 6-FAM (Integrated DNA Technologies, Coralville, Iowa, USA) and a second primer annealing to different sites of the Mu or *E. coli *genome (see Additional file 3). PCR reactions containing 50 ng of input DNA template, 10 pmoles of each primer and 1× Taq polymerase Master Mix (Qiagen) were placed in a thermal cycler (PTC-200 MJ Research) for 22 cycles. PCR products were purified using a Qiaquick PCR Purification Kit^® ^(Qiagen) and analyzed using a genetic analyzer (3130XL; Applied Biosystems) and interpreted using the analysis software GeneMaker (Version 1.5; SoftGenetics LLC).

### ChIP

SJG3, SJG3 *Δfis *and SJG3 *Δhns *strains were grown to mid-log phase (OD_600 _approximately 0.6) in LB at 30°C, and the temperature-sensitive Mu repressor was inactivated at 42°C for 40 minutes. Cells were cross-linked by 1% (v/v) formaldehyde (37% (v/v) solution; Fisher Scientific) at room temperature for 30 minutes, and excess formaldehyde was quenched with 125 mM glycine at room temperature for 5 minutes. The cells were collected by centrifugation, washed twice with Tris-buffered saline (pH7.5), and resuspended in lysis buffer containing 50 mM Tris-HCl, pH7.5, 1 mM EDTA, 100 mM NaCl, 4 mg/ml lysozyme (Roche) and protease inhibitors (1 tablet per 10 ml buffer) (Roche). The suspension was incubated at 37°C for 30 minutes, followed by the addition of same volume of 2× IP buffer (100 mM Tris-HCl (pH7.5), 1 mM EDTA, 200 mM NaCl and 2% (v/v) TritonX-100). The lysate was sonicated (Vibra Cell^®^, model VC 505l; Sonics) seven times for 20 seconds each in an ice bath to shear the chromatin DNA. The size of DNA resulting from ultrasonication was 300 to 1000 bp, with an average size of 500 bp. The cell debris generated was removed by centrifugation at 21,000 *g *for 15 minutes at 4°C, and the supernatant was used as 'input' samples for immunoprecipitation. To immunoprecipitate c-myc-MuB-DNA or MuA-DNA, 5 μg of anti-c-myc antibody (9E10; Covance) and anti-MuA serum [28], respectively, were added to 500 μl of input samples (MuA anti-serum was purified using Montage Antibody Purification Kit with PROSEP-A media from Millipore, in accordance with their instruction manual.) No-antibody controls ('mock' samples) were included as well. After overnight incubation at 4°C, 50 μl Dynabeads Protein A (Invitrogen) were added to the samples. Following a 5 hour incubation at 4°C, the beads were washed twice with immunoprecipitation (IP) buffer (50 mM Tris-HCl, pH7.5, 140 mM NaCl, 1 mM EDTA and 1% (v/v) TritonX-100), once with wash buffer I (50 mM Tris-HCl, pH7.5, 500 mM NaCl, 1 mM EDTA and 1% (v/v) TritonX-100), once with wash buffer II (10 mM Tris-HCl, 250 mM LiCl, 1 mM EDTA and 1% (v/v) TritonX-100) and once with TE buffer (10 mM Tris-HCl, pH8.0, 1 mM EDTA). After aspirating off the TE buffer, the beads were suspended in 150 μl of elution buffer (50 mM Tris-HCl, pH8.0, 10 mM EDTA and 1% (v/v) SDS) and incubated for 45 minutes at 65°C. The supernatant was carefully transferred to a new tube and heated to 65°C for at least 6 hours to reverse protein-DNA crosslinking. Afterwards, 150 μl of TE buffer were added and the mixture was first treated with 0.5 μg/ml RNaseA (Sigma) at 37°C for 30 minutes and then with 100 μg/ml proteinase K (Sigma) at 55°C for 2 hours. The sample was then purified (Qiaquick PCR Purification Kit^®^; Qiagen).

### Normal PCR

An aliquot (1 μl) of ChIP or mock sample was added to 5 μl of 10× Expand Long Template buffer 1 (Roche), 1.5 μl of dNTP (10 mM each), 1.5 μl of each primer (10 μM), 0.75 μl of Expand Long Template Enzyme Mix (Roche), and 38.5 μl H_2_O. The samples were heated for 2 minutes at 94°C, after which they underwent 30 cycles of 15 seconds at 94°C, 15 seconds at 55°C and 30 seconds at 68°C, followed by incubation for 7 minutes at 68°C (PTC-200 MJ Research).

### Real-time qPCR

Mu insertions within the Mu genome or the *E. coli *chromosome were measured by PCR reactions using packaged Mu DNA as template and different primers pairs, all of which had a common primer annealing within the Mu right end, with the second primer annealing within the region of interest (see Additional file 3). Enrichment of MuB-bound Mu genome DNA or *E. coli *chromosome DNA was measured in ChIP samples by PCR reactions using specific primer pairs annealing within these DNAs. The real-time qPCR conditions were: 50 ng of Mu DNA or 1 μl of ChIP or mock-ChIP DNA, 10 μl SYBR master mix (Qiagen; includes dNTPs, enzyme and buffer), 0.4 μl of each primer (10 μM) and 8.2 μl of double distilled H_2_O. The reactions were held for 10 minutes at 95°C followed by 40 cycles of 15 seconds at 95°C and 1 minute at 60°C (7900HT; Applied Biosystems). Three independent biological replicates were tested, and for each biological replicate three independent technical replicates were performed.

Primer efficiencies were determined as follows: MuR was linked to pUC18f and the various primers were linked to pUC19r; the pUC primers anneal to the pUC19 plasmid and amplify a common 180 bp fragment. The PCR products were purified (Qiaquick PCR purification Kit^®^; Qiagen) and used as templates for qPCR in the following reaction: 12.5 μl SYBR mix (Qiagen), 0.75 μl of MuR (10 μM) paired with various primers (10 μM), 1 μl of template (10^-5 ^ng/μl) and 10 μl of ddH_2_O. The reactions were held for 10 minutes at 95°C, followed by 40 cycles of 15 seconds at 95°C and 1 minute at 60°C (7900HT; Applied Biosystems). The template input amount was controlled by qPCR using the primer pair pUC19f and pUC19r. The primer efficiency of Mu1 was set as 1, and the efficiencies of the other primer pairs were normalized to that of Mu1.

Mu copy number was calculated as follows. Genomic DNA was isolated from SJG3 at 0, 20 and 40 minutes after prophage induction (Wizard Genomic DNA Purification Kit, Promega). The purified DNA were diluted to 1 ng/μl and used as template in the following qPCR reactions: 12.5 μl SYBR mix (Qiagen), 0.75 μl of primer pairs within Mu1, 1 μl of genomic DNA and 10 μl of ddH_2_O. The reactions were held for 10 minutes at 95°C, followed by 40 cycles of 15 seconds at 95°C and 1 minute at 60°C (7900HT; Applied Biosystems). The template input amount was controlled by qPCR using primer pairs within L25. The Ct differences between Mu1 and L25 represent the copy number of Mu. When the copy number of Mu at 0 minutes was set as 1, the copy number at 40 minutes was calculated to be 18. This number is an average of three technical repeats.

## Competing interests

The authors declare that they have no competing interests.

## Authors' contributions

JG designed the experiments, performed the research and analyzed the data. ZL assisted with the research. RMH conceived of the study, participated in its design and coordination and helped to draft the manuscript. All authors read and approved the final manuscript.

## Supplementary Material

Additional file 1 **Figure S1**. (**a**) Lysis profiles of various Mu lysogens. All cultures were grown at 30°C to an OD_600 nm _of 0.6, shifted to 42°C for 40 minutes for phage induction, and shifted down to 37°C until lysis. 0 indicates time of temperature shift-up. Δ*hns *strain did not lyse naturally, so lysis was induced by addition of chloroform. Phage titers from all three strains were similar to those from the parent strain MP1999, yielding around 3 to 5 × 10^8 ^pfu/ml. ChIP samples were prepared at 40 minutes for all SJG3-derived strains. (**b**) Western blot of MP1999 with anti-MuB antibodies at indicated times after temperature shift-up. Click here for file

Additional file 2 **Figure S2**. Moving average AT-content of Mu genome. The window for average AT-content (%) calculation is 100 bp and the moving step is 20 bp. The value of AT content at a given position represents the average AT content of 100 bp DNA starting from that position. The graph was derived from data in [20]. Click here for file

Additional file 3 **Table S1**. Sequence of primers used in this study. Click here for file
