# Supplementary figures and images for: Immunity of replicating Mu to self-integration: a novel mechanism employing MuB protein
Source: Mob DNA. 2010 Feb 1;1:8. doi: 10.1186/1759-8753-1-8 (PMC2837660; doi:10.1186/1759-8753-1-8)

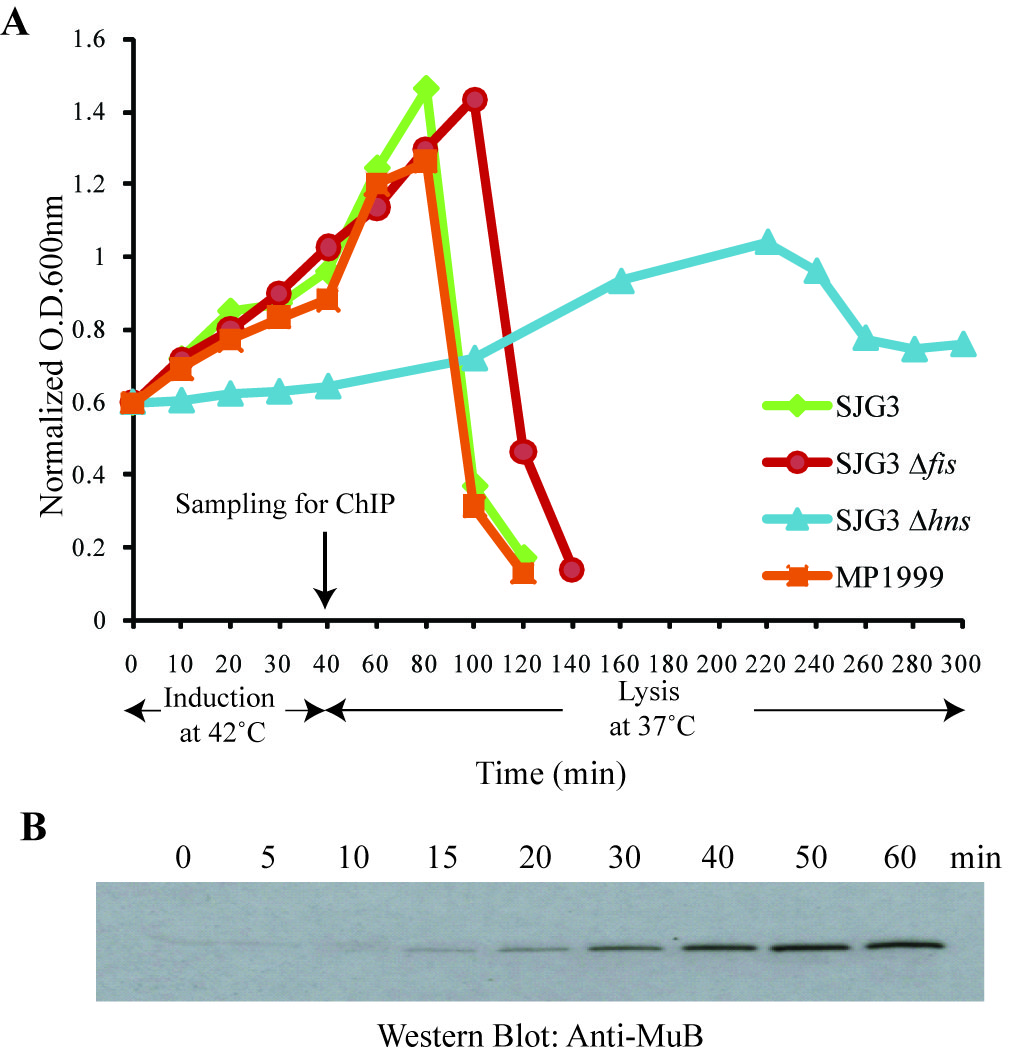

Supplement: Additional file 1 — Figure S1. (a) Lysis profiles of various Mu lysogens. All cultures were grown at 30°C to an OD600 nm of 0.6, shifted to 42°C for 40 minutes for phage induction, and shifted down to 37°C until lysis. 0 indicates time of temperature shift-up. Δhns strain did not lyse naturally, so lysis was induced by addition of chloroform. Phage titers from all three strains were similar to those from the parent strain MP1999, yielding around 3 to 5 × 10^8^ pfu/ml. ChIP samples were prepared at 40 minutes for all SJG3-derived strains. (b) Western blot of MP1999 with anti-MuB antibodies at indicated times after temperature shift-up. [file 1759-8753-1-8-S1.JPEG]

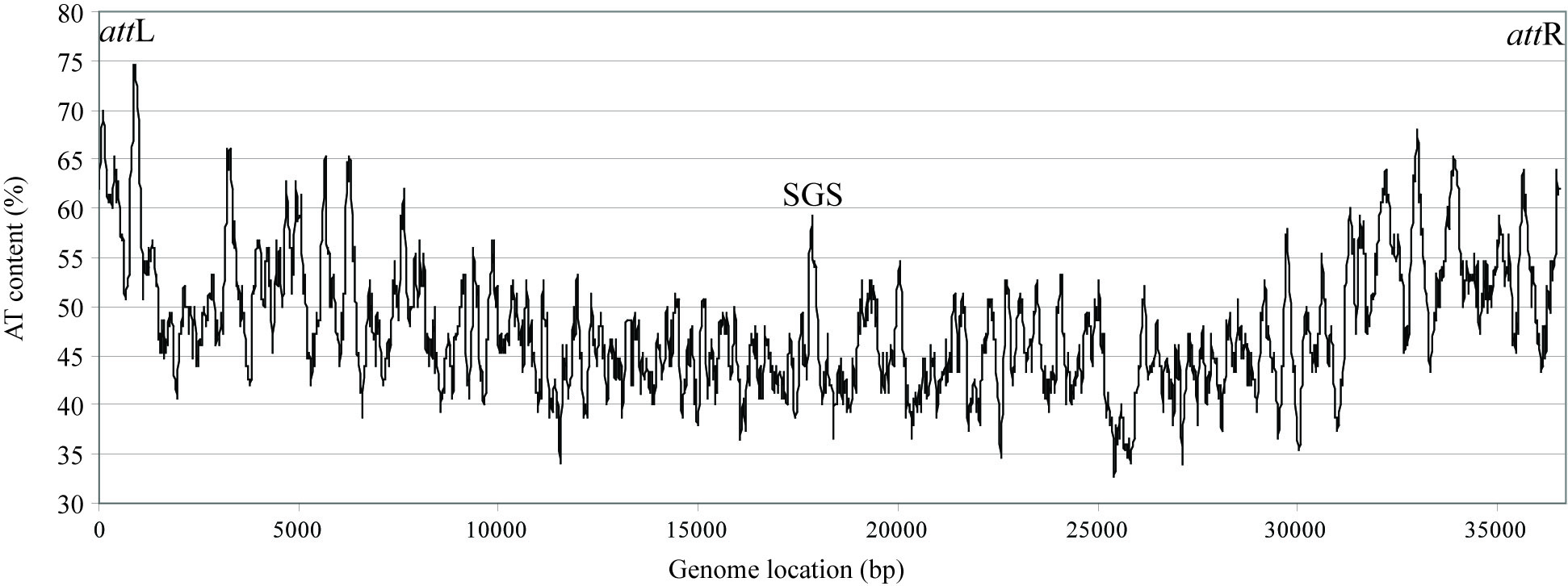

Supplement: Additional file 2 — Figure S2. Moving average AT-content of Mu genome. The window for average AT-content (%) calculation is 100 bp and the moving step is 20 bp. The value of AT content at a given position represents the average AT content of 100 bp DNA starting from that position. The graph was derived from data in [20]. [file 1759-8753-1-8-S2.JPEG]
