# Supplementary material for: Immunity of replicating Mu to self-integration: a novel mechanism employing MuB protein
Source: Mob DNA. 2010 Feb 1;1:8. doi: 10.1186/1759-8753-1-8 (PMC2837660; doi:10.1186/1759-8753-1-8)
Supplement: Additional file 3 — Table S1. Sequence of primers used in this study. [file 1759-8753-1-8-S3.DOCX]

**Table S1**. Primers used.

| **Primer Name** | **sequence** |
| --- | --- |
|  |  |
| ***for fragment analysis and qPCR*** | |
| MuR-FAM | 6FAM / CGC TTT CGC GTT TTT CGT GC |
| MuR | CGC TTT CGC GTT TTT CGT GC |
| Mu1f | TGT ATT GAT TCA CTT GAA GTA CGA AAA AAA CCG GGA |
| Mu2f | TGT GGC GAT GTT CAA TGC CAG AAC |
| Mu3f | ACC ATC AGT GCC GTG GTG TAT CTT |
| Mu4f | GAT CTG GCA CGC AGT ATT ACC ACC GAT TAT GG |
| Mu5f | CAT TCA GCG CCT GCA TTC TGA ACA |
| Mu6f | AGA TGA CAG ACC GCC AAG ACG AAT |
| Mu7f | ATC ACG AGA TAA CGA TGA ACG CGA TTA AGC |
| yidPf | ATG ATC TAC AAA AGC ATT GCG GAG CGG |
| rfaSf | ATG ACT ATT TAT TTT ATA AAT TGG GTT GCA GAT TAT G |
| ahpFf | ATC AAC GTG AAC TTT CAG TGC GCC |
| Mu1r | AAA GCC GTA TAA CAA CCA TGA ACC AGC TAA |
| Mu2r | AAT GCA ACA GGT GCC AGA CAT TCC |
| Mu3r | ACT GGC AGT AAA GCA AAT CGT CGC |
| Mu4r | TGA GTA CTG GAG AAA GAA AGT GAA AGG AAG A |
| Mu5r | TGA CAC CGG TTA CAG CTT CCA TCA |
| Mu6r | TGG CGA CGA GAC TAC AAC ACG AAA |
| Mu7r | TGA AGC GGC GCA CGA AAA ACG CGA AA |
| yidPr | TCG TTG ATT TGA ATC CGT AAC TGG CTG |
| rfaSr | AGT ATT TCT TAG AAG CAA AAC TTT ATG ACA |
| ahpFr | AAC GAA ATC ACC GAT CGC AAC GTG |
| L25f | ACT ATT ATT CAG GGC GGC ACG TCT |
| L25r | TCG AGG AAT CGA CCA TAC TGA GCA |
| L10f | TCA GGT GTT ATC ACA GGA CTG GCT |
| L10r | GCG GTC TGC GTT GGA TTG ATG TTT |
| L5f | GTG GCA ACA CCT TTA TGT TTG GCG |
| L5r | TTT GAT CCC ATC CGG CAG ATC GTT |
| L1f | GTT GGA ATT GTC AGG CAG TTA AGG C |
| L1r | TGT TCT GCC AAT GTT ATG CCG CTG |
| L0f | ATC AGC AGC GGG AAC ATA ATC GCT |
| L0r | ATT CAA CTG TTA ACC AAC GGC GGC |
| R0f | AAA TCG TCC GGA ATC GCT TTC AGC |
| R0r | TGG TGT TCT CGC TGA TCA CTG TCT |
| R1f | AGC GTG GTA ATG GCG AAC AGG TAT |
| R1r | TGA AAG ACG CGC AGA CTC GTA TCA |
| R5f | CAA CAA CGG TCA CAT GCT GCG TAT |
| R5r | TTT CTC ATC CCA CTT GGC GTA GGT |
| R10f | TGA TCA TCC GGC TTT CTT CTG GGT |
| R10r | TCT TCG ACA AGG CGG TGT TCA GTT |
| R25f | GAA CTT GAT ACT TCT TCT CCA GGT CT |
| R25r | TGA TGA ATC CTC GGC AAA TGA GCG |
|  |  |
| ***for adding c-myc tag to MuB in lysogen*** | |
| MuB-cat-sacBf | GCC ATT TAA TTA ACG TTT AAA CAA AAT TTA ATT ACG AGG TTA TTC AGA TG ATC AAA GGG AAA ACT GTC CAT AT |
| MuB-cat-sacBr | GTT GCC ATT TTT ATA TTT CGG GCC ATC ATA ATT TCT TCT CCT TCT TA TGT GAC GGA AGA TCA CTT CG |
| cat-sacB-cmycMuBf | GTT GCC ATT TTT ATA TTT CGG GCC ATC ATA ATT TCT TCT CCT TCT TA ATT ACG CAG CAG CGT TGA AAT AT |
| cat-sacB-cmycMuBr | GCC ATT TAA TTA ACG TTT AAA CAA AAT TTA ATT ACG AGG TTA TTC AGA TG TCC GGT TCT GCT GCT AGT GGT |
|  |  |
| ***for deleting fis*** |  |
| fis-knf | CGA AAA TTT TGC GTA AAC AGA AAT AAA GAG CTG ACA GAA CTA TGG TGT AGG CTG GAG CTG CTT C |
| fis-knr | CCG AGT AGC GCC TTT TTA ATC AAG CAT TTA GCT AAC CTG AAT TAC ATA TGA ATA TCC TCC TTA |
|  |  |
| ***for deleting hns*** |  |
| hns-knf | CGC CGC TGG CGG GAT TTT AAG CAA GTG CAA TCT ACA AAA GAT TAG TGT AGG CTG GAG CTG CTT C |
| hns-knr | ACC TCA ACA AAC CAC CCC AAT ATA AGT TTG AGA TTA CTA CAA TGC ATA TGA ATA TCC TCC TTA |
|  |  |
| ***for primer efficiency*** | |
| pUC19f | TGC GCA ACG TTG TTG CCA TT |
| pUC19r | AAC ACT GCG GCC AAC TTA CT |
